# Evidence available and used by the Food and Drug Administration for the approval of orphan and nonorphan drugs

**DOI:** 10.1093/haschl/qxaf057

**Published:** 2025-03-18

**Authors:** Amanda J Koong, Veronica L Irvin, Aditya Narayan, Sujin Song, Robert M Kaplan

**Affiliations:** Department of Health Policy and Management, Fielding School of Public Health at the University of California at Los Angeles, Los Angeles 90095, USA; McGovern Medical School at UTHealth Houston, Houston 77030, USA; College of Health, Oregon State University, Corvallis 97331, USA; Stanford University Schools of Medicine and Business, Stanford 94305, USA; Clinical Excellence Research Center, Stanford University School of Medicine, Stanford 94304, USA; Clinical Excellence Research Center, Stanford University School of Medicine, Stanford 94304, USA

**Keywords:** FDA approval, rare diseases, orphan drugs, drug regulation, randomized clinical trials, clinical trial transparency

## Abstract

There are substantial financial incentives to develop orphan drugs for rare diseases, but concerns about the quality and volume of supporting evidence have emerged. We compare evidence used to evaluate orphan and nonorphan drugs approved by the Food and Drug Administration (FDA) between 2016 and 2023. This retrospective cross-sectional analysis utilizes FDA data on approvals and study information from ClinicalTrials.gov to compare characteristics of studies relevant to orphan and nonorphan drugs approved between 2016 and 2023. Of the 368 total drugs approved, 50% were orphan drugs. The FDA-approved drugs based on significantly fewer studies for orphan (1.5 studies/drug) compared to nonorphan (2.4 studies/drug). Additionally, a significantly lower proportion of studies were completed before FDA approval for orphan drugs (25% vs 41%). Orphan drugs were significantly less likely to be evaluated in randomized clinical trials (RCTs) (34% vs 63%). Of these RCTs, there were significantly fewer completed before approval (40% vs 54%) and that had results posted (35% vs 53%). There was a significant difference in the available evidence for orphan and nonorphan drugs. As new legislation like Cures 2.0 is developed, it is critical to examine the balance between an expedited approval timeline and the standard of clinical evidence.

## Introduction

Diseases are currently classified as rare if they affect less than 200 000 individuals in the United States.^1^ The US National Institute of Health (NIH) has identified approximately 7000 rare diseases.^2^ These conditions collectively account for 6%-7% of all diseases^1^ and affect an estimated 25 million individuals in the United States.^3^

Prior to 1983, pharmaceutical companies had not devoted much attention to rare diseases, primarily due to high research and development costs paired with a limited market size.^3^ In response, Congress passed the Orphan Drug Act in 1983 to promote the development of treatments for rare diseases through financial incentives to pharmaceutical companies. Specifically, the Food and Drug Administration (FDA) waived new drug applications, granted a market exclusivity of 7 years, gave companies tax credits (25% of research and development costs), and allocated more funds for rare disease research grants.^3^ The NIH has steadily increased funding for rare disease research since 2013.^2,4^ Following the passage of the Orphan Drug Act in 1983, orphan drug designations approximately doubled every 10 years from the 1980s through the 2000s and nearly tripled from 2000 to 2010. Orphan drugs now provide an attractive niche opportunity for many biopharmaceutical companies, and some propose that profits are equal to or greater than those of nonorphan drugs.^5,6^ They allow for more rapid development, lower research development expenses, other tax incentives, higher approval rates, lower risk of generic competition, and premium pricing.^5^

The passage of the 21st Century Cures Act allowed for the use of nontraditional trials, analysis methods, and surrogate biomarkers.^7,8^ This shift ultimately decreased the number of randomized clinical trials (RCTs) used in the evaluation.^7,9^ Recent literature aims to evaluate the impact of changing standards, particularly the implications for patients with rare diseases, an already vulnerable population.^10-12^

While promoting the development of orphan drugs has the potential for important clinical benefits, it also has potential drawbacks. For instance, Daniel et al.^13^ reported significant patterns of pharmaceutical companies that submit applications to the FDA for orphan drugs. Once their products are approved, they promote broad off-label usage to a wide patient market while such drugs continue to benefit from orphan drug protections and exclusivity benefits. More broadly, following the approval of the Orphan Drug Act, 69% of orphan drugs had changes to the safety labels after approval, 15% of which were associated with severe safety events.^14^

The Cures 2.0 Act^15^ is a bipartisan effort to build on the original 21st Century Cures Act to further drive medical innovation and patient-centered healthcare. The proposal includes automatic Medicare coverage for FDA-designated breakthrough medical technologies.

As Congress deliberates Cures 2.0, it is essential to consider the implications for the current approval standards, particularly in vulnerable populations, such as patients with rare diseases. Specifically, we believe Sections 301 “Report on collaboration and alignment in regulating digital health technologies,” Sec. 304 “Increasing use of real world evidence,” and Sec. 309 “Post-approval study requirements for accelerated approval” are important to consider in light of our findings.

This study compares the quantity and type of studies registered to ClinicalTrials.gov for orphan and nonorphan drugs approved by the FDA from 2016 to 2023.

## Methods

This cross-sectional study retrospectively examined data from the FDA Novel Drug Approvals from 2016 through 2023. Novel drug approvals are new molecular entities that have not previously been approved by the FDA. Thus, approvals and trials for additional indications of previously approved drugs were not included. Data regarding the approval date, studies used in approval, orphan drug status, priority review, accelerated approval, breakthrough, and fast-track designation were recorded. Drugs were included if the FDA designated them as “nonorphan” or “orphan.” The FDA considers orphan drugs to treat conditions that impact less than 200 000 people in the United States.^16,17^

For each novel drug approval, we used the National Library of Medicine ClinicalTrials.gov website to identify all registered studies for the respective active ingredient and clinical indication. We systematically downloaded all standardized fields, which included but were not limited to whether study results were posted, the conditions tested, primary and secondary outcomes, sponsor(s), participant sexes, participant ages, enrollment numbers, study design, start date, completion date, and the date that results were first posted. Studies that had a status of “terminated” or “withdrawn” were excluded. All study phases were included. We merged this data with data from the FDA's website that reports drug approval date, the number of trials used in drug approval, whether a drug a issued a priority voucher, and whether a drug was granted the following designations: orphan drug, accelerated approval, breakthrough therapy, priority review, fast track, and qualified infection disease product. All designations were based on the reports by the FDA for each novel drug and the disease indication submitted for approval. All data were collected by December 2023.

### Analysis

First, we standardized our data collection by having all five coauthors code data for the same five drugs per year to ensure consistency across drugs and years. Then, each coauthor coded 1 or 2 years of drugs approved by the FDA. Analyses were performed with R version 4.2.0. A Welch two-sample *t*-test was applied to compare differences between nonorphan and orphan drugs. The significance level was set at *P* < 0.05.

## Results

From 2016 to 2023, 368 drugs were approved. Of these, 183 (50%) were designated as “nonorphan drugs” and 185 drugs (50%) were designated as “orphan drugs.”

As indicated in Table 1, the median study enrollment for studies of nonorphan drugs was 150 in comparison to 65 participants for orphan drugs. The interquartile range of enrollment was 407 and 142 for nonorphan and orphan drugs, respectively. We explored enrollment numbers as a potential predictor of study-level outcomes, such as whether a study was randomized or had results reported (Table S1). However, when orphan drug status was held constant, the effects of the number of study enrollees were not statistically significant (Table S1).

**Table 1.** Characteristics of nonorphan vs orphan drugs approved by FDA between years 2016 and 2023.

	Nonorphan drugs	Orphan drugs
Total # of drugs	183 (50%)	185 (50%)
Total # of trials in ClinicalTrials.Gov	3293 (63%)	1933 (37%)
Median trial enrollment (participants)	150	65
Interquartile range of enrollment	407	142
Mean # of trials in FDA approval	2.4	1.5
# of studies receiving priority review	89 (49%)	152 (82%)
Accelerated approval	13 (7%)	54 (29%)
Breakthrough therapy designation	28 (15%)	79 (43%)
Fast-track designation	49 (27%)	91 (49%)
Median # of total trials registered in ClinicalTrials.gov per drug	12	8
Mean # of total trials registered in ClinicalTrials.gov per drug	21	13.8
Mean # of trials completed before approval	6.3	3.3
Mean # of trials completed after approval	13.6	9.6
Mean # of trials with results posted before approval	1.9	0.6
Mean # of trials with results posted after approval	4.4	1.9
Mean # of RCT per drug	9	4.4
Study design		
Nonrandomized	333 (10%)	352 (18%)
Observational	298 (9%)	226 (12%)
Observational cohort	111 (3%)	26 (1%)
Randomized	1752 (53%)	600 (31%)
Single group assignment	741 (23%)	671 (35%)
Other	58 (2%)	58 (3%)
Study status		
Active, not recruiting	368 (11%)	366 (19%)
Completed	1617 (49%)	590 (31%)
Not yet recruiting	199 (6%)	160 (8%)
Recruiting	868 (26%)	696 (36%)
Other	105 (3%)	89 (5%)
Unknown	136 (4%)	32 (2%)
RCT only		
Mean # of trials completed before approval	4.2	2
Mean # of trials completed after approval	4.1	1.9
Mean # of trials with results posted before approval	1.5	0.4
Mean # of trials with results posted after approval	2.9	0.7
Funding type		
Industry	1871 (57%)	1104 (57%)
Industry/other	116 (4%)	95 (5%)
NIH	59 (2%)	39 (2%)
NIH/Other	25 (0.8%)	30 (2%)
Other	1222 (37%)	665 (34%)
Route of administration		
Injection	51 (28%)	70 (38.5%)
Intravenous	16 (9%)	11 (6%)
Ophthalmic	6 (3%)	1 (0.5%)
Oral	81 (44%)	82 (44%)
Subcutaneous	7 (4%)	4 (2%)
Topical	7 (4%)	2 (1%)
Other^a^	15(8%)	13 (7%)

^a^Catheter installation, intrathecal, inhalation, intramuscular, intravitreal, nasal, and vaginal.

The mean number of total studies registered in ClinicalTrials.gov to evaluate each drug was 21 for nonorphan drugs and 13.8 for orphan drugs. The mean number of RCTs registered in ClinicalTrials.gov per drug is 9 (nonorphan drugs) and 4.4 (orphan drugs). Overall, 53% of total registered studies for nonorphan drugs were randomized, compared to 31% for orphan drug studies. Funding for nonorphan and orphan drugs was distributed similarly, with approximately 57% of studies funded via industry and 2% of studies funded via NIH.

On average, trial results for nonorphan drugs were posted 607.5 days after study completion in comparison to 410.1 days for orphan drugs (Table 2). This difference was not statistically significant (*P* = 0.106) (Table 2). Differences in the mean number of adverse postmarket safety warnings for orphan and nonorphan drugs were nonsignificant (*P* = 0.34).

**Table 2.** Comparisons between nonorphan and orphan drugs.

	Nonorphan mean	Orphan mean	*t* value	Degrees of freedom	*P* value
# of total studies per drug	21.0	13.8	2.8	345.9	*P* < 0.005
# of studies used in approval	2.4	1.5	4.8	261.9	*P* < 0.001
Mean # of adverse post market safety warnings	0.75	0.69	−0.95	344.2	*P* = 0.34
Proportion of studies completed before approval	0.41	0.25	5.1	359.8	*P* < 0.001
Proportion of studies completed after approval	0.49	0.64	−4.1	364.6	*P* < 0.001
Proportion of studies with results before approval	0.14	0.07	3.6	294.6	*P* < 0.001
Proportion of studies with results after approval	0.25	0.14	4.6	310.2	*P* < 0.001
Days between completion to results posted	607.5	410.1	1.6	434.9	*P* = 0.106
Proportion of RCTs	0.63	0.34	9.3	284.6	*P* < 0.001
Proportion of RCTs completed before approval	0.54	0.4	2.9	232.4	*P* < 0.005
Proportion of RCTs completed after approval	0.4	0.53	−2.7	223.2	*P* < 0.01
Proportion of RCTs with results posted	0.53	0.35	4.1	233.3	*P* < 0.001
Proportion of RCTs with results before approval	0.2	0.14	1.8	253.7	*P* = 0.0669
Proportion of RCTs with results after approval	0.33	0.22	2.9	249.7	*P* < 0.005

We found a significant difference in the total studies registered per drug (*P* < 0.005) between nonorphan (21.1 studies/drug) and orphan (13.8 studies/drug) (Table 2). The proportion of studies completed before approval differed significantly (*t* = 5.1, *P* < 0.001) between nonorphan (41% of studies) and orphan drugs (25% of studies). These proportions were calculated for each drug individually and then averaged for nonorphan and orphan drugs, respectively. As seen in Table 2, the proportion of studies with results posted to ClinicalTrials.gov before drug approval also differed significantly between nonorphan and orphan drugs (*t* = 3.6, *P* < 0.001).

**Figure 1.**
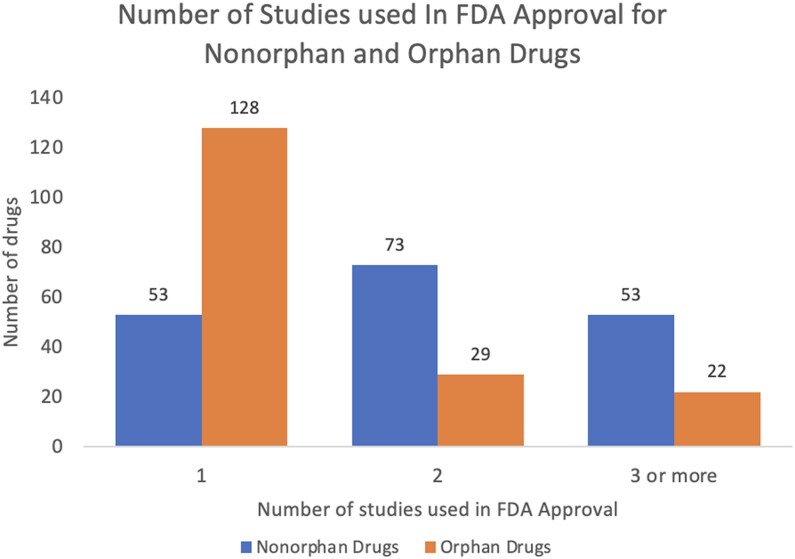
Number of studies used in FDA approval for each drug approved between 2016 and 2023 by nonorphan vs orphan drugs.

There was a significant difference (*P* < 0.001) in the mean number of studies used in each drug approval (2.4 for nonorphan drugs and 1.5 for orphan drugs) (Table 2). More orphan drugs (128) than nonorphan drugs (53) were approved on the basis of a single study (Figure 1). In contrast, more nonorphan drugs than orphan drugs were approved on the basis of multiple studies.

For each drug, we also analyzed the proportion of trials that were prospectively registered as RCTs and found that 53% of RCTs for nonorphan drugs had results posted, compared to only 34% of RCTs for orphan drugs. There was a significant difference (*P* < 0.001) in RCTs that were completed before drug approval for nonorphan (54%) and orphan (40%) drugs (Table 2).

To further explore relationships between the drug classes and the conditions they treat, we examined the relationship between oncology drug status, orphan drug status, and drug-level outcomes. Broadly, whether a drug was approved for cancer was a significant predictor of most drug-level outcomes when holding orphan drug status constant. Similarly, orphan drug status was still a significant predictor of these outcomes while holding oncology drug status constant (Table S2).

**Figure 2.**
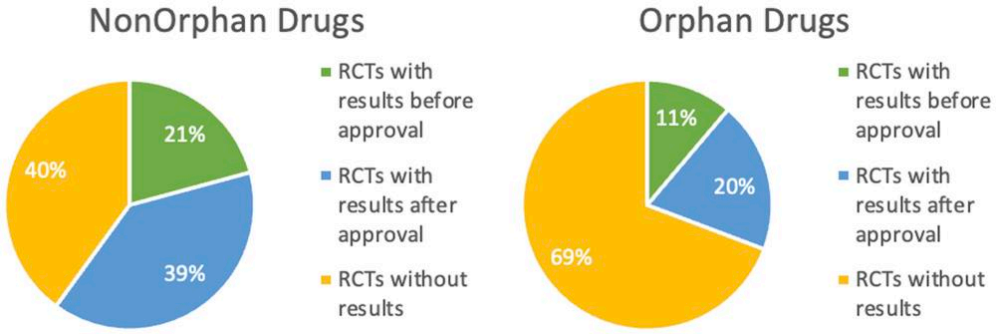
Percentage of RCTs registered in ClincialTrials.gov prior to FDA approval with results posted never, before, or after approval.

Twenty percent of RCTs evaluating nonorphan drugs posted results before approval, compared to 14% of RCTs in orphan drugs (Figure 2). While results were posted significantly more often for RCTs evaluating nonorphan drugs than orphan drugs (*P* < 0.001), differences were nonsignificant for RCTs with results posted before approval (*P* < 0.067) (Table 2).

## Discussion

The body of evidence available to evaluate orphan drugs differs significantly from that of nonorphan drugs. Between 2016 and 2023, orphan drugs, on average, had been evaluated in fewer total studies, had results reported less frequently, and had been assessed less frequently using an RCT. There were also fewer orphan drug studies completed before the approval date, indicating that less information was available to both the FDA and the public to evaluate the product. Furthermore, significantly fewer studies were used to justify approval, and there were fewer participants in each study compared to their nonorphan drug counterparts. While the body of evidence for orphan drugs is weaker, they also benefit from increased rates of priority review, accelerated approval, breakthrough therapy, and fast-track designations.

Overall, these results are consistent with the literature examining orphan drug evaluations. Chen et al.^18^ studied 87 clinical trials used in the approval of 72 novel orphan drugs and similarly found that orphan drugs often lack robust clinical trial support and are frequently approved on the basis of lower-quality evidence. Sasinowski et al.^19^ reviewed noncancer orphan drugs and found similar reports that the FDA did not require conventional levels of evidence in order to approve such drugs and granted extraordinary leniency for noncancer orphan drugs. Similarly, Michaeli et al.^10^ compared cancer drug approvals for rare and common cancers, concluding that evidence for rare cancers depended on less robust trials that risked overestimating therapeutic benefits. Dupont and Van Wilder found that orphan drug designation led to higher Belgian reimbursement rates than their nonorphan counterparts despite weaker clinical evidence.^20^

Orphan drug studies report results at lower rates compared to nonorphan drugs. However, for the studies that did report results, they were released 197 days sooner on average than their orphan drug counterparts. This finding could be due to increased pressure from rare disease advocacy groups with a vested interest in the results of specific trials. Of note, the average days from study completion to results reported for both orphan (607.5 days) and nonorphan (410.1 days) studies exceeds the FDA's 365-day mandate. The 2017 Food and Drug Administration Amendments Act (FDAAA) introduced penalties for failing to report results within the specified timeline, with the ability to fine up to $10 000 per day. As of 2024, the FDA had not collected any fines, while uncollected potential fines were estimated to total nearly 63 billion dollars,^21^ a figure that is 7.6 times higher than the FY2023 FDA budget of $8.3 billion.^22^ Daval et al.^23^ outlined potential reasons the FDA is not incentivized to collect fines and potential steps the FDA can take to enforce regulations in the future. They suggest that the FDA may rely on pharmaceutical companies to invest in the research and development of novel therapies. Souring such relationships or disincentivizing them to pursue these avenues may lead to fewer therapeutic developments for patients.

We found a significant relationship between cancer drug status and drug-level outcomes, such as the proportion of studies completed before approval, even when controlling for orphan drug status. This finding aligns with work from Vaghela et al.,^24^ which describes significant differences in evidence used in FDA approval for oncologic vs nononcologic drugs. Like orphan drugs, cancer drugs are often targeted at a small patient population. As such, it is reasonable that both characteristics are predictors of the same outcomes.

The RCTs are typically considered the most rigorous study design, and while they have drawbacks, they are often cited as the gold standard for evaluating new treatment modalities.^25^ Orphan drug studies had disproportionately fewer RCTs registered to ClinicalTrials.gov. By the nature of rare diseases, recruitment for large and well powered trials is often more difficult, as the patient population is smaller and geographically dispersed. Furthermore, bioethical concerns make it difficult to use placebo or usual care control groups. As such, the FDA allows flexibility in trial design and other data sources, such as surrogate endpoints and real-world evidence (RWE), when reviewing orphan drugs. However, studies show that although there is an increased reliance on postapproval studies, there is no significant increase in the frequency of conducted and completed postapproval studies.^26,27^ Historically, real-world data and RWE are more accepted for orphan drugs than nonorphan drugs, given the limitations of evaluating rare diseases.^28^ Recently, there have been calls and attempts to develop a hybrid trial methodology, synthesizing the strengths of traditional RCTs and observational study designs to produce regulatory-grade RWE.^29^ Others detail statistical considerations needed to reconcile RWE and RCTs.^30,31^

As such, we aimed to target our analysis to only RCTs, where we found that orphan RCTs still report results significantly less often in comparison to nonorphan RCTs. This is an important finding because there are fewer randomized trials to evaluate orphan drugs. Of the existing RCTs, there should be a greater push to publish these results, ensuring greater transparency of important information about a vulnerable population.

## Limitations

Our results should be interpreted in the context of several limitations. First, our analysis depends on the quality of data reported to ClinicalTrials.gov. Principal investigators can complete studies and publish results in medical journals without directly uploading their results to ClinicalTrials.gov. To that end, all other variables reported in ClinicalTrials.gov are self-reported by principal investigators, thus leaving room for discrepancies. Furthermore, findings such as low proportions of RCTs and even lower proportions of completed RCTs could be a function of underreporting to ClinicalTrials.gov.

Additionally, the scope of our study is limited to novel drug approvals, meaning new molecular entities that have not previously been approved by the FDA. There are various significant differences in the approval of drugs for their original indications compared to their secondary indications.^32^ Because we focus on novel drug approvals, a portion of indications are not captured. This limits the generalizability of our conclusions.

Furthermore, while the FDA reports which studies are officially used in their approval process, it is impossible to know how the data are used, including the quality of the data generated in each study. Pharmaceutical companies may present trial data to the FDA that are not available to the public. Finally, only information about the products approved by the FDA is available to the public. We do not have information about products that were evaluated but ultimately rejected by the FDA. Thus, we cannot draw conclusions about what characteristics are associated with approvals compared to those that are rejected.

Our findings may be relevant to Sections 301, 304, and 309 of the proposed Cures 2.0 Act.^15,33^ While it is important to allow for diverse study designs for orphan drugs, the FDA must still ensure there is sufficient evidence for safety and efficacy. Additionally, Cures 2.0 should consider enhanced postmarketing surveillance and postapproval study requirements, given the increased reliance on non-RCT data. Finally, given the lack of transparency and reporting compliance to Clinicaltrials.gov, it is important to ensure that Cures 2.0 defines and provides resources to enforce these reporting standards.

## Conclusion

Overall, our findings support the need for a broader discussion regarding the standards used to approve orphan drugs and their potential implications.^34^ Orphan drugs serve vulnerable populations with limited treatment options and limited knowledge of the disease process. It is understandable that there are fewer RCTs conducted in these populations. A smaller patient population limits the feasibility of some clinical trial designs, including randomized studies. However, the FDA has a responsibility to ensure that the results of completed orphan drug RCTs are available to the public, at least at similar rates to nonorphan drug trials. As new legislation, such as Cures 2.0, is written and passed, it is critical to ensure that results from trials are published, particularly for orphan drugs, which intrinsically have fewer trials than nonorphan drugs.

## Supplementary Material

qxaf057_Supplementary_Data
