# Supplementary material for: Evidence available and used by the Food and Drug Administration for the approval of orphan and nonorphan drugs
Source: Health Aff Sch. 2025 Mar 18;3(4):qxaf057. doi: 10.1093/haschl/qxaf057 (PMC11970248; doi:10.1093/haschl/qxaf057)
Supplement: qxaf057_Supplementary_Data [file qxaf057_supplementary_data.zip › Supplemental Tables.docx]

**Supplemental Tables**

Supplemental Table 1: Study Level Characteristics, Controlling for Number of Enrollees

| Outcome |  | Orphan Drug Status | Number of Enrollees |
| --- | --- | --- | --- |
| Study Results Reported | Odds Ratio | 0.647 | 0.99 |
|  | P value | P<0.001 | P = 0.213 |
| Study Randomized | Odds Ratio | 0.802 | 0.99 |
|  | P value | P<0.001 | P = 0.1 |
| Completed Before Approval | Odds Ratio | 0.495 | 1.00 |
|  | P value | P<0.001 | P = 0.355 |
| Results Posted On or Before Approval | Odds Ratio | 0.536 | 0.99 |
|  | P value | P<0.001 | P=0.595 |

Supplemental Table 2: Drug Level Characteristics, Controlling for Oncology drug status

| Outcome |  | Orphan Drug Status | Oncology drug status |
| --- | --- | --- | --- |
| Proportion of studies completed before approval | Odds Ratio | 0.89 | 0.76 |
|  | P value | P<0.001 | P<0.001 |
| Proportion with results posted before approval | Odds Ratio | 0.95 | 0.92 |
|  | P value | P<0.001 | P<0.001 |
| Proportion of RCTs | Odds Ratio | 0.77 | 0.85 |
|  | P value | P<0.001 | P<0.001 |
| Priority Review | Odds Ratio | 3.49 | 4.35 |
|  | P value | P<0.001 | P<0.001 |
| Accelerated Approval | Odds Ratio | 3.47 | 17.42 |
|  | P value | P<0.001 | P<0.001 |
| Breakthrough Therapy | Odds Ratio | 3.24 | 3.72 |
|  | P value | P<0.001 | P<0.001 |
| Fast Track Designation | Odds Ratio | 2.58 | 0.94 |
|  | P value | P<0.001 | P = 0.819 |
